# Variability of gene expression profiles in human blood and lymphoblastoid cell lines

**DOI:** 10.1186/1471-2164-11-96

**Published:** 2010-02-08

**Authors:** Josine L Min, Amy Barrett, Tim Watts, Fredrik H Pettersson, Helen E Lockstone, Cecilia M Lindgren, Jennifer M Taylor, Maxine Allen, Krina T Zondervan, Mark I McCarthy

**Affiliations:** 1 Genetic and Genomic Epidemiology Unit, Wellcome Trust Centre for Human Genetics, Oxford, UK; 2 Oxford Centre for Diabetes, Endocrinology and Metabolism, University of Oxford, Oxford, UK; 3 Genomics Laboratory, Wellcome Trust Centre for Human Genetics, Oxford, UK; 4 Bioinformatics Core, Wellcome Trust Centre for Human Genetics, Oxford, UK

## Abstract

**Background:**

Readily accessible samples such as peripheral blood or cell lines are increasingly being used in large cohorts to characterise gene expression differences between a patient group and healthy controls. However, cell and RNA isolation procedures and the variety of cell types that make up whole blood can affect gene expression measurements. We therefore systematically investigated global gene expression profiles in peripheral blood from six individuals collected during two visits by comparing five of the following cell and RNA isolation methods: whole blood (PAXgene), peripheral blood mononuclear cells (PBMCs), lymphoblastoid cell lines (LCLs), CD19 and CD20 specific B-cell subsets.

**Results:**

Gene expression measurements were clearly discriminated by isolation method although the reproducibility was high for all methods (range ρ = 0.90-1.00). The PAXgene samples showed a decrease in the number of expressed genes (P < 1*10^-16^) with higher variability (P < 1*10^-16^) compared to the other methods. Differentially expressed probes between PAXgene and PBMCs were correlated with the number of monocytes, lymphocytes, neutrophils or erythrocytes. The correlations (ρ = 0.83; ρ = 0.79) of the expression levels of detected probes between LCLs and B-cell subsets were much lower compared to the two B-cell isolation methods (ρ = 0.98). Gene ontology analysis of detected genes showed that genes involved in inflammatory responses are enriched in B-cells CD19 and CD20 whereas genes involved in alcohol metabolic process and the cell cycle were enriched in LCLs.

**Conclusion:**

Gene expression profiles in blood-based samples are strongly dependent on the predominant constituent cell type(s) and RNA isolation method. It is crucial to understand the differences and variability of gene expression measurements between cell and RNA isolation procedures, and their relevance to disease processes, before application in large clinical studies.

## Background

The advent of microarray technology has led to genome-wide interrogation of transcript abundance. Numerous studies have characterised variation in human gene expression associated with cell and tissue type, environmental conditions or disease and these have led to a better understanding of biological pathways. For clinical purposes, gene expression signatures have been useful to classify tumours [1,2], to identify diagnostic markers [3] or patient groups that benefit from therapies [4] and to understand infectious disease processes [5].

Alongside genome-wide association studies and upcoming sequencing studies, there is increasing interest in obtaining large-scale "omics" data from large biobanks and sample collections, including gene expression, proteomic and metabonomic profiling. These biobanks will rely on easy sample collection and handling using robust methodologies and sample storage over a prolonged time period. While the downstream gene expression profiling techniques using microarrays are very reliable for large-scale investigations, there are still challenges prior to microarray analysis including the choice of a relevant sample type and RNA and cell isolation method. Blood-based samples will continue to be one of the most readily available sources for gene expression studies in large-scale investigations. Several strategies - ranging from PAXgene (which captures RNA profiles of all cell types in whole blood and has no complex cell isolation procedures prior to RNA isolation) to the creation of lymphoblastoid cell lines (LCLs) comprising a transformed single cell type - have been developed. Other isolation methods attempt to generate a subset of cell types such as peripheral blood mononuclear cells (PBMCs) by the use of Ficoll or lymphocyte subsets using magnetic beads.

Peripheral blood contains a variety of cell types including erythrocytes, granulocytes, lymphocytes, monocytes, natural killer cells and platelets. In PBMCs, several cell types including neutrophils, basophils, eosinophils, platelets, reticulocytes and erythrocytes are depleted. Because each of the contributing cell types expresses a unique gene expression signature relating to its function, the relative proportions of the cell types affect the gene expression profile [6]. In addition, the relative proportions of the cell types can change rapidly following disease-related or inflammatory responses. Clearly, this variability may confound the interpretation of gene expression differences between control and disease groups.

Investigating gene expression profiles in homogeneous cell populations, such as T or B lymphocytes, that have a potential as markers of infection or disease, might resolve such variability and could have greater diagnostic power than whole blood profiles [6,7]. The extraction of more homogeneous cell populations, however, which is often laborious and difficult to standardize, involves manipulation of the cells and may influence the expression profiles [6-9].

One source that is used extensively to study genetic influences on expression [10-12] or to investigate host responses to pathogens [5] is LCLs. The substantial advantage of LCLs over whole blood is that the impact of environmental influences or other cell types on expression is much reduced, allowing - in theory - a more powerful investigation of genetic influences. However, LCLs are transformed and cultured under artificial conditions and may not represent the natural gene expression state *in vivo *due to a large percentage of pauciclonality combined with widespread monoallelic expression [13,14].

In order for gene expression profiling in blood to become a reliable and reproducible tool in large-scale investigations, a better understanding of intra- and interindividual variability comparing used methods is needed. Several studies have shown that the PAXgene system using whole blood samples results in higher variability of gene expression profiles and a decrease in expressed genes compared to PBMC-based methods [6-9]. However, Whitney et al. observed a higher variability of gene expression profiles in individuals with disease than among healthy individuals in blood, indicating the feasibility of using gene expression profiling in blood for disease detection and diagnosis [6].

Several studies have examined the variability and gene expression signatures in whole blood and PBMCs in healthy individuals using different cell and RNA isolation procedures [6-8,15-21]. Only one study investigated gene expression signatures of purified T- and B-lymphocytes and granulocytes [9] and little work has been done to explore differences in gene expression profiles from LCLs and B cell subsets. A comparison between the variability and gene expression signature of LCLs to other blood-based subtypes is of particular relevance, given the extent to which this sample type is currently being used for expression Quantitative Trait Loci studies [10-12].

In the present study, we investigated variability and consistency in gene expression profiles between five of the most common post venipuncture methods of cell and RNA isolation: whole blood (PAXgene (PAX)), PBMCs, Epstein-Barr virus (EBV) transformed LCLs, CD19-specific B-cells subsets (B-cell CD19), CD20-specific B-cells subsets (B-cell CD20). Using samples from six individuals collected during two visits, we evaluated the differences and concordances of global gene expression profiles, the biological and technical variability seen in these approaches, cell-type specific gene expression signatures and their relevance to large-scale biobanking initiatives.

## Results and Discussion

### High reproducibility between visits and high variability between methods

**Figure 1:**
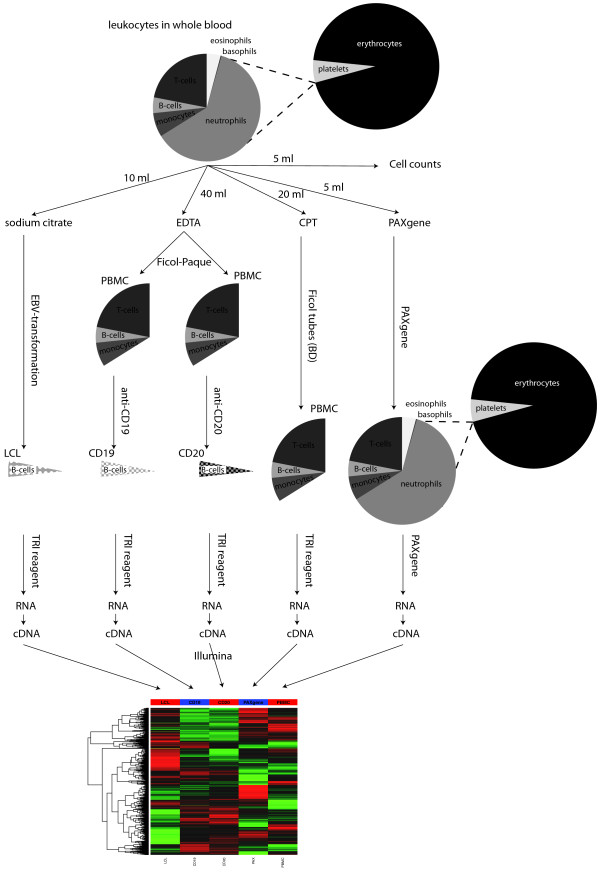
**Study design**. We obtained gene expression profiles of five different post venipuncture methods of cell and RNA isolation. The pie charts illustrate the different cellular composition of the five methods whereas the arrows show the laboratory processes.

To determine the effect of the cell and RNA isolation method on global gene expression profiling, gene expression profiles for 56 out of 60 samples were successfully generated on Illumina Ref 6 arrays (see Methods). The study design is shown in Figure 1. Four samples failed gene expression profiling probably due to low yield or low quality (see Additional file 1). Remaining samples were checked using unsupervised analysis (see Methods).

To evaluate the reproducibility, variability and signal-noise ratio between the five cell and RNA isolation methods, we examined variability between visits and probes. To explore visit variability (intra-individual), we calculated Spearman rank sum correlations between the two visits across all probes after applying two common probe filters (standard deviation (SD) and detection score). The correlations ranged between 0.86-0.92 for all probes, 0.83-0.90 for probes with SD > 0.5 and 0.90-1.00 for probes with detection score > 0.95 indicating a higher reproducibility between visits using the detection score as a probe filter (Table 1). These correlations (same individual) were higher than between random individuals. Although PAX showed a high reproducibility between visits, it provided significantly fewer detected probes (N = 8,783, 19%) than the other isolation methods (range = 10,672-12,122 probes, 23-26%; P < 1*10^-16^) (Table 1). The percentage of variable probes (SD > 0.5) in PAX, however, was significantly higher (73%) compared with the other methods (52%-65%; P < 1*10^-16^).

**Table 1:** Variability and reproducibility after applying two common probe filters (detection score >0.95 and SD > 0.5) for each RNA and cell isolation method.

RNA and cell isolation method	No. of probes with SD > 0.5	No. of probes with detection score >0.95	Spearman correlation range across replicates* Mean(range)	Spearman correlation range across random individuals Mean(range)
**PAX**	34,012	8,783	0.96 (0.96-0.98)	0.93 (0.90-0.95)

**PBMC**	27,987	11,834	0.98 (0.90-0.99)	0.96 (0.93-0.97)

**LCL**	24,311	11,865	0.99 (0.96-1.0)	0.96 (0.96-0.97)

**Bcell CD19**	24,229	12,122	0.99 (0.98-0.99)	0.97 (0.93-0.96)

**Bcell CD20**	30,342	10,672	0.96 (0.93-0.98)	0.95 (0.96-0.97)

* Spearman correlations are calculated between two visits for each matched or random individual for each method. Spearman correlations are based on detected probes.

Consistent with our findings, previous studies found a reduction of detected probes, lower gene expression signals and increased inter-individual variability as compared to PBMCs [7,8]. Because the main differences between PAX and PBMCs are the depletion of erythrocytes and reticulocytes from the latter, it is assumed that these differences are related to the abundant mRNA expression of members of the hemoglobin gene family [8,22-25]. Previous studies have shown that depletion of globin mRNA resulted in an increased number of detected probes, a decrease of variability and improved detection sensitivity for mRNAs from non-reticulocyte cell types [8,22-27] but we did not specifically test this option in the present study.

**Figure 2:**
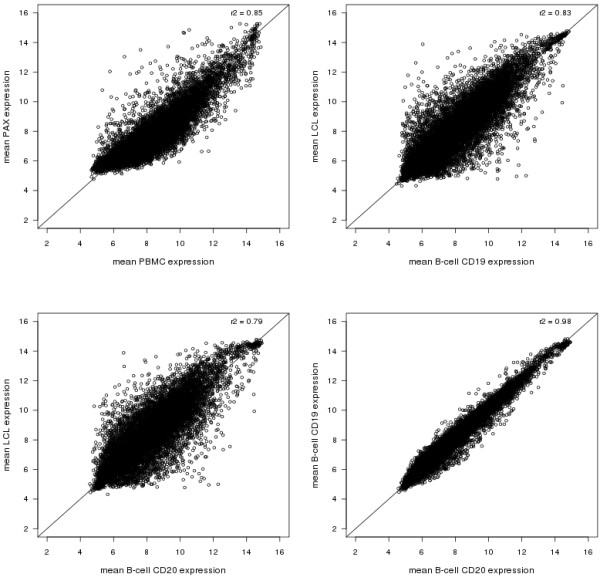
**Scatterplots of mean expression levels across individuals**. Gene expression levels are averaged for the two visits of the overlapping detected probes.

We next calculated the mean expression values across individuals and visits for each overlapping detected probe between four pairs of cell and RNA isolation methods with (partly) corresponding or closely related cell types to visualize inter-individual variability: i) PAX and PBMCs, ii) LCLs and B-cell CD19, iii) LCLs and B-cell CD20, and iv) B-cell CD19 and B-cell CD20 (Figure 2). The PAX expression levels are decreased but more variable than the PBMC expression levels (ρ = 0.85). The LCL expression levels are of similar magnitude as the B-cell CD19 or B-cell CD20 expression levels (ρ = 0.83 and ρ = 0.79 respectively) but lower than the correlation between B-cell CD19 and CD20 (ρ = 0.98).

Variation in expression profiles between different isolation methods and visits can originate from both biological and technical sources. Inter-individual biological variation can arise from variation such as genetic variation, cellular composition, ethnicity, sex, genotype-environment interactions or physiological variation such as time of the day at which a sample was taken, diet and stress. The latter would also contribute to variability between multiple visits [6,7,9-11,28]. Technical variation can be caused by the different steps of the experiment such as sample preparation, isolation of cellular components, labelling, hybridisation and time to analysis [6,7,9].

We found high correlations between visits for each method (ρ = 0.96-0.99) but lower correlations between different methods (ρ = 0.79-0.98) suggesting that the cell or RNA isolation method has a larger impact on the gene expression profile than the variability between visits. The decreased correlations between LCLs and B-cell CD19 or B-cell CD20 might have resulted from the controlled *in vitro *conditions of the LCLs or the B-cell purifications.

Methods that involve much post-processing provide less variability but these manipulations might alter gene expression patterns from those *in vivo*. The intrinsic and extrinsic factors play a key role in choosing the most preferable study design. In genetic studies, homogeneous cell populations - in which extrinsic factors are minimized compared to *ex vivo *samples - are more useful whereas for biomarker detection whole blood samples capturing *in vivo *conditions more accurately could be more informative.

### Gene expression profiles are dependent on cell and RNA isolation method

**Figure 3:**
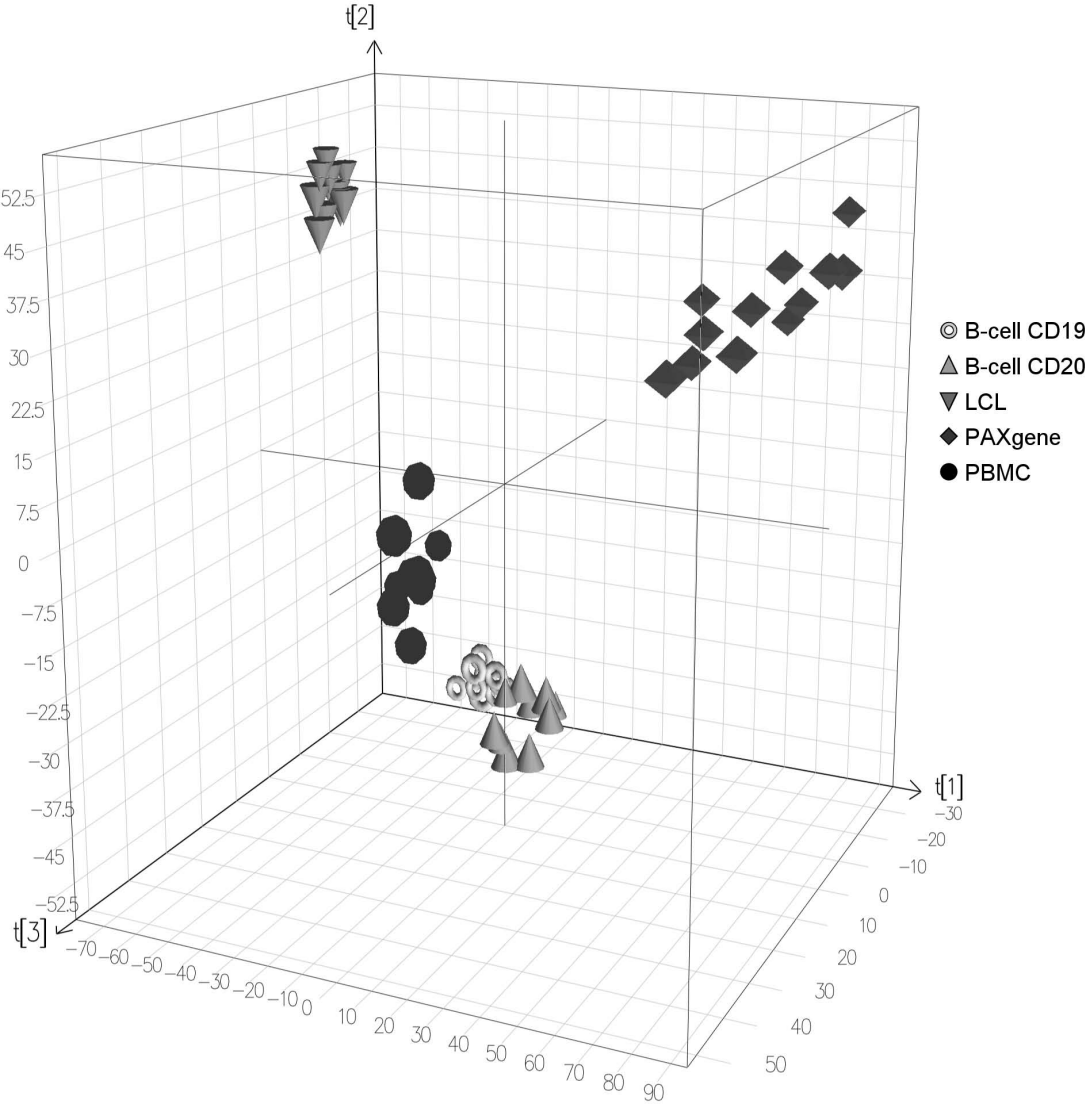
**Principal components analysis of the samples**.

To explore and visualise sources of variation in this dataset, we clustered a subset of 7,305 probes that were expressed in all 56 samples with a detection score >0.95 using principal components analysis (PCA) and hierarchical clustering methods. Figure 3 shows that PCA with three components separated the samples according to the five methods. The first two components in the PCA separated the PBMCs, B-cell CD19, B-cell CD20 from PAX and LCLs explaining 70% of the variance. The third component discriminated the PBMCs from B-cell CD19 and B-cell CD20 explaining 9.8% of the variance. Notably, B-cell CD19 and B-cell CD20 samples were clustered together.

We computed Partial Least Squares Discriminant Analysis (PLS-DA) models for each isolation method to examine sets of genes whose transcripts are responsible for separating the methods. For each model, we extracted the variable weights of the expression probes, ranked these variable weights and selected the 5% highest and 5% lowest ranked expression probes. Table 2 shows genes that were strongly up- or down-regulated in the PLS-DA models.

**Table 2:** Genes that were strongly up- or down-regulated for each cell and RNA isolation method.

RNA and cell isolation method	Up-regulated genes	Down-regulated genes
**PAX**	*SLC25A37*, *TYROBP*, *WDR40A*	*RPL31*, *RPS27L, RPL26*

**PBMC**	*NKG7*, *GZMB, SH2D1A*	*CD70*, *TNFRSF13C*,*TNFRSF13B*

**LCL**	*FSCN1*, *CD70*, *TNFSF9*	*FCRL3*, *RASGRP2*,*TYROBP*

**Bcell CD19**	*BANK1*,*FAM129C*,*FCRL3*	*LGALS3*, *WDR40A*, *FSCN1*

**Bcell CD20**	*BANK1*,*FAM129C*, *FCRL3*	*LGALS3*, *WDR40A*, *FSCN1*

These ten subsets of expression probes were then analyzed for statistical enrichment of Gene Ontology (GO) terms for Biological processes using all 7,305 expressed probes as a background list. The up-regulated probes in LCLs and the down-regulated probes of the B-cell CD20 samples (with an overlap of 50% of probes) revealed an enrichment of alcohol metabolic process (GO:0006066, False Discovery Rate (FDR) P = 2.0*10^-7 ^and FDR P = 0.03) (see Additional file 2).

**Figure 4:**
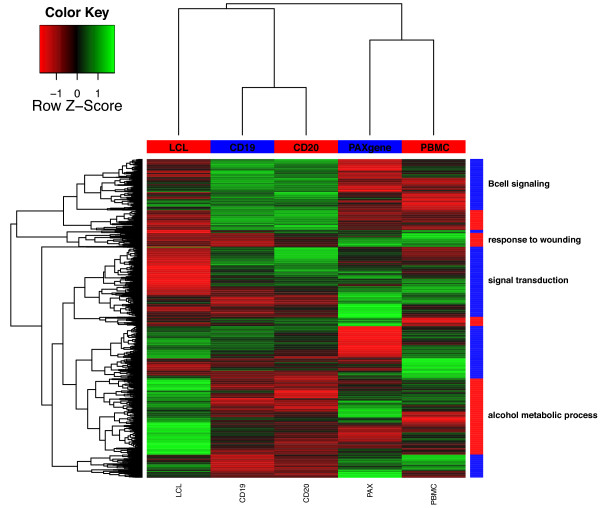
**Hierarchical clustering of 2,072 probes with 5% lowest and 5% highest PLS variable weights expressed across all 56 samples**.

The GO terms "response to wounding" (GO:0009611) and "signal transduction" (GO:0007165) were enriched in the down-regulated probes of the LCLs (FDR P = 0.02, FDR P = 1.2*10^-8^) and the up-regulated probes of the PAX (FDR P = 0.002; FDR P = 0.001) and PBMCs (FDR P = 6.4*10^-4^; FDR P = 8.7*10^-11^). Hierarchical clustering of the variable weights of the 2,072 down- and up-regulated expression probes of all five methods resulted in clustering of transcripts according to these GO terms (Figure 4). To examine the concordance of gene expression measurements across methods, we extracted 1,952 expression probes that showed variable weights between -0.01 and 0.01 for all methods. GO analysis showed an enrichment of "secretion by cell" (GO:0032940: FDR P 3.2*10^-3^) and "antigen presentation and processing" (GO:0048000, FDR P = 0.02).

### Gene expression differences between isolation methods are associated with cellular composition and B-cell manipulation

**Figure 5:**
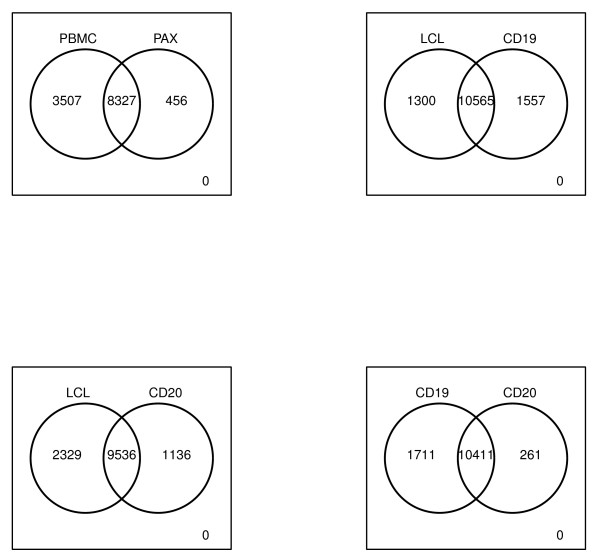
**Venn diagrams of the number of detected probes between A) PAX and PBMCs B) B-cell CD19 and LCLs C) B-cell CD19 and LCLs D) B-cell CD19 and B-cell CD20**.

Because PLS-DA analysis only gives an overview of variation for probes across all methods, we refined our GO analysis by making pair-wise comparisons of closely related isolation methods focusing on i) uniquely detected probes and ii) overlapping detected probes that were significantly differentially expressed between two methods (Figure 5). In this analysis, we ranked the probes on significance and then selected 5% of the most significant probes that showed an at least three-fold change.

In the PAX-PBMC comparison, 456 probes were detected in PAX but not in the PBMCs and 3,507 probes vice versa. For the uniquely detected probes in the PBMCs and PAX, none of the GO terms was significantly enriched after FDR correction. We found 374 (4.5%) probes differentially expressed between PAX and the PBMCs (Table 3). These probes showed an enrichment of "gas transport" (GO:0015669) containing genes *CA2*, *HBD *and *HBQ1 *in PAX (Table 4). The GO term "Macromolecule biosynthetic process" (GO:0009059) was most significantly enriched in the PBMCs containing 33 genes (including *GYPC*, *RPL26L1*, *EEF1B2*, *RPS27A*, *MTIF2*) encoding proteins such as ribosomal proteins, translation initiation and elongation factors.

**Table 3:** The number of differentially expressed probes between cell and RNA isolation methods after FDR correction.

RNA and cell isolation method	No. probes	5% top hits with three fold change		
		
		+	-	%
**PAX - PBMC**	8,327	268	106	4.5

**LCL - Bcell CD19**	10,565	358	138	4.7

**LCL - Bcell CD20**	9,536	336	136	4.9

**Bcell CD19 - CD20**	10,411	13	18	0.3

**Figure 6:**
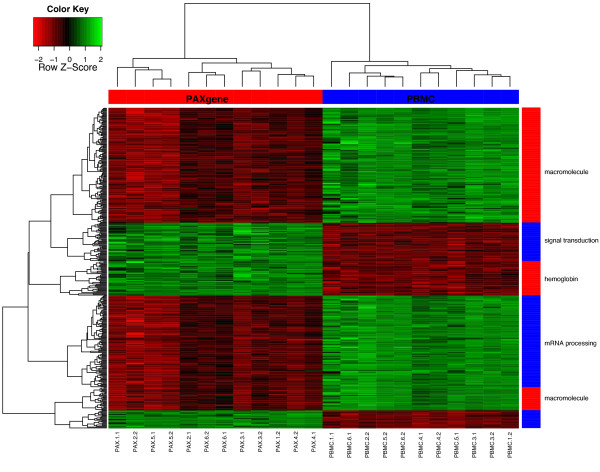
**Hierarchical clustering of 374 differentially expressed probes for the PAX and PBMCs**.

**Figure 7:**
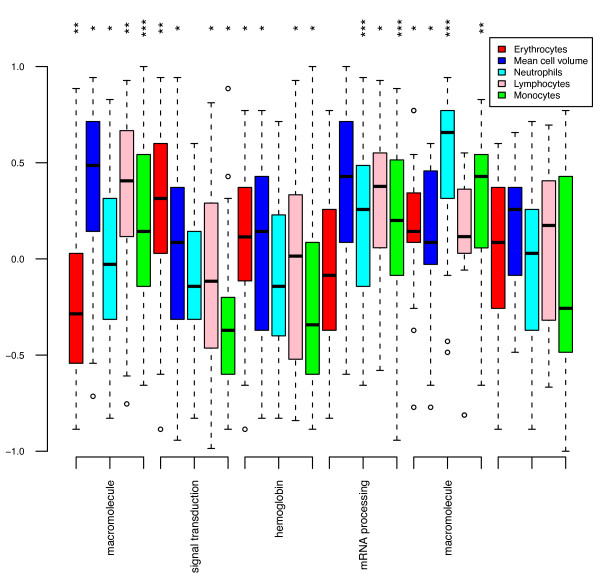
**Clusters of differentially expressed probes are correlated with several parameters of blood counts (number of neutrophils, number of lymphocytes, number of erythrocytes, number of monocytes and mean cell volume)**. Open circles indicate outlying values in PAX. Significance levels are indicated at the top: * p-values ≤ 0.05 and > 10^-5^,** p-values are ≤ 10^-5 ^and >10^-10^,*** p-values are ≤ 10^-10^.

These findings suggest that the gene expression differences between the PBMCs and PAX are caused by the differences in cellular composition; gas transport is specific for erythrocytes and translation and transcription are physiological responses more important in lymphocytes and monocytes than in granulocytes [6,9]. To explore whether enrichment is derived from the most abundant cell types in the sample, we clustered the differentially expressed transcripts in six groups and correlated the transcripts in each group to the cell counts in whole blood (Figure 6). All three groups up-regulated in the PBMCs showed significant positive correlations with monocyte counts (ρ = 0.20, p = 2.2*10^-16^; ρ = 0.20, p = 2.7*10^-14^; ρ = 0.29, p = 5.9*10^-7^) and platelets (ρ = 0.39, p < 10^-16^; ρ = 0.42, p < 10^-16^; ρ = 0.47, p = 6.6*10^-7^). Only one of these groups was correlated with lymphocytes (ρ = 0.36, p = 1.3*10^-7^) whereas the two other groups of up-regulated genes were significantly positively correlated with neutrophil count (ρ = 0.20, p = 1.3*10^-14^; ρ = 0.50, p = 4.1*10^-11^). Three groups of probes were up-regulated in PAX containing probes targeting "hemoglobin" and "signal transduction" genes and the latter was significantly positively correlated with erythrocyte count (ρ = 0.25, p = 2.5*10^-6^) and mean cell volume (ρ = 0.25, p = 8.3*10^-5^) (Figure 7).

To prevent the difficulties of cell type mixtures, B-cell specific methods have been developed. To investigate to which extent B-cell specific methods differ from each other, we compared gene expression measurements of LCLs with B-cell CD19 and B-cell CD20. For the B-cell CD19 and CD20, 1,557 and 1,136 probes were uniquely expressed compared with the LCLs (Figure 5). In both B-cell CD19 and CD20 the GO term (GO:0009611) "response to wounding" (FDR P = 3.8*10^-9 ^and FDR P = 1.5*10^-11^) was most significantly enriched. This category contained B-cell specific genes encoding complement pathway components (*CD40lg*, *CD180*), interleukins (*IL-6*), chemokine receptors (*CCR2*, *CCR3*), immunoglobulin receptors (*FCER1G*) and members of the toll-like receptor family (*TLR4*, *TLR8*) (see Additional file 3).

In the LCLs, 1,300 and 2,329 probes were uniquely expressed as compared to B-cell CD19 and CD20 (Figure 5). These probes showed an enrichment of the GO term "Cell cycle phase" (GO:00022403, FDR P = 0.005 and FDR P = 1.5*10^-4^) and included genes involved in mitosis (*CCNB1*, *CENPF*, *PBK, TTK*). Between the B-cell CD19 or B-cell CD20 and LCL samples, 496 (4.7%) and 472 (4.9%) probes were differentially expressed. In both B-cell CD19 and CD20 samples, glycolysis GO terms were enriched whereas cell cycle GO terms were only enriched in B-cell CD19 (Table 4). The GO analysis suggested an increased rate of glycolysis and cell cycle in LCLs as compared with B-cells. B lymphocytes found in the peripheral circulation are in a non-proliferative state and require stimulation with an antigen to enter the cell cycle. Hollyoake et al. showed that infection with EBV causes the infected cells to activate the cell-division cycle [29].

**Table 4:** Enrichment of GO terms among differentially expressed probes between different cell and RNA isolation methods.

Go Term (Biological Process)	Description	No. genes (%)	P	FDR P
**PAX versus PBMC**				

GO:0015669	Gas transport	6 (2.3)	2.1*10^-5^	0.01

GO:0009059	Macromolecule biosynthetic process	33 (12.9)	8.3*10^-5^	0.03

**B-cell CD19 versus LCL**				

GO:0000278	Mitotic cell cycle	24 (6.2)	5.9*10^-5^	0.02

GO:0006066	Alcohol metabolic process	23 (5.9)	3.6*10^-5^	0.02

**Bcell CD20 versus LCL**				

GO:0006066	Alcohol metabolic process	22 (6.5)	1.1*10^-5^	0.02

## Conclusion

Gene expression profiling of blood is a valuable tool for diagnostics in a wide range of diseases, particularly those involving the immune system and cancer. Before peripheral blood or cell lines can be used in large cohorts to characterise differences between a patient group and healthy controls, it is important to understand the underlying biological and technical factors that contribute to the gene expression measurements. Our results give insight into the variability and characterisation of biological differences between post venipuncture methods including LCLs, purified B-cells (CD19 and CD20), PBMCs and whole blood samples for global gene expression profiling. The number of expressed genes as well the gene expression measurements differ significantly between different isolation techniques. Although the PAXgene system is suitable for large-scale gene expression profiling, particularly in large epidemiological and biobank studies where immediate sample processing is not always practical, the PAX samples showed a decrease in the number of expressed genes and lower gene expression values with higher variability compared to the PBMCs. Although whole blood samples contain more cell populations with different relative proportions than PBMCs, expression profile differences between the two isolation methods are also likely to be (partly) caused by the abundance of globin mRNA. Additional steps in the PAX protocol involving globin reduction could improve sensitivity and variability of this sample type relative to other isolation methods [8,22-27].

The up-regulated probes in PBMCs showed significant positive correlations with the number of monocytes, lymphocytes and neutrophils, whereas the down-regulated probes were correlated with the number erythrocytes and mean cell volume. Our comparison between B-cell subsets and LCLs showed that the correlations between the expression levels of detected probes were much lower compared to the two B-cell isolation methods. More specifically, enrichment of inflammatory response genes in the B-cell CD19 and CD20 may represent the lack of external stimuli of the *in vitro *controlled conditions in LCLs or the manipulation of the B-cell CD19 and CD20. Conversely, the enrichment of glycolysis and cell cycle genes in LCLs might appear as adaptation to the *in vitro *cell transformation of B-cells to LCLs and might reflect indefinite LCL propagation.

In this study, we used two positive selection approaches -using incubation of PBMCs with anti-CD19 or anti-CD20- to purify B-cell populations. A potential limitation of these approaches is the activation of cell surface receptors that might alter gene expression. Further studies of gene expression profiles of other more recently developed B-cell selection methods using a negative selection approach should further improve our understanding of gene expression variability in blood [30].

Some of these cell and RNA isolation methods are widely used in large-scale clinical studies; indeed, PAXgene is a likely to be a favoured method for general whole blood expression profiling in samples stored in large biobanking facilities. It is, however, crucial to consider what effect the choice of a specific RNA isolation procedure has on the ability to detect certain gene expression profiles and their likely relation to the disease of interest.

## Methods

### Subjects and blood samples

Blood was taken from six healthy volunteers seen twice in two weeks. All volunteers were Caucasian, healthy, not on medication and non-fasted. Complete blood counts were determined by standard procedures and included: cell counts (white cells, erythrocytes, leukocytes, platelets, neutrophils, lymphocytes, monocytes, eosinophils and basophils), hemoglobin, hematocrit and erythrocyte indices (mean corpuscular volume, mean corpuscular hemoglobin and mean corpuscular hemoglobin concentration). All subjects fell within normal ranges for the major cell populations.

For each individual, five different post venipuncture methods were performed (Figure 1). B Lymphocytes from 10 ml of blood were isolated by tubes with sodium citrate. LCLs were generated by EBV-mediated transformation and cells were grown for eight weeks.

For the isolation of CD19 and CD20 B-cells, 40 ml whole blood from EDTA tubes was collected and PBMCs were isolated by using a Ficoll-Paque™ gradient (Amersham). CD19 and CD20 B-cells were prepared by positive selection from the PBMCs by incubation with magnetic anti-CD19 or CD20 mAb-coated microbeads (MACS, Miltenyi Biotec). For the isolation of PBMCs from whole blood, BD Vacutainer^® ^CPT Mononuclear Cell Preparation Tubes (Becton and Dickinson) were used. Total RNA was isolated from 5 ml of whole blood samples with the PAXGene Blood RNA system (QIAGEN) and samples were left at room temperature for 24 hours before processing according to manufacturer's instructions.

Only two people at a time were sampled on any one day for logistical reasons. After blood draw standard protocols were followed for cell isolation, transformation or RNA extraction. With the exception of the PAXgene samples all RNA was isolated using TRI™ reagent (SIGMA) and resuspended in RNase free water.

This research was carried out in compliance with the Helsinki Declaration, and was carried out under ethical approvals granted to the MolPAGE project by Oxfordshire Research Ethics Committee B (05/Q1605).

### Pre-processing of microarray data

After RNA had been isolated successfully for 59 samples, RNA quantity was measured using a Nanodrop ND-1000 Spectrophotometer to give the yield and a 260/280 ratio. Agilent Bioanalyser Lab-on-a-chip RNA chips were also run for each sample to check the quality by calculating RNA Integrity Number (RIN) scores. 500 ng of total RNA was labelled using the TotalPrep™ RNA Amplification Kit (Ambion Inc.). For each of the five methods, samples from two visits of an individual were measured on the same Beadchip and samples from each individual were measured on a maximum of three Beadchips to maximise biological reproducibility and minimise technical variability.

Expression profiling was completed using Human-6 version 2 Sentrix BeadArrays (Illumina Inc.) which contains 48,702 unique probes covering 28,567 RefSeq annotated transcripts. Arrays were hybridised with labelled cRNA material and scanned according to manufacturer's instructions. The resultant data were parsed with the software package BeadStudio (Illumina Inc.) to produce raw intensity values for all probes. Signal was checked for quality using hybridisation and labelling controls internal to each array and subtracted for background within the statistical scripting environment, R v2.4.1 [31]. Signal was transformed and normalised using the variance stabilization algorithm as implemented in the vsn2 [32] Bioconductor [33] package. Transformed and normalised signal distributions for each sample were investigated with unsupervised analysis to identify outliers.

### Data quality, probe mapping and filtering

Gene expression profiling was successful for 56 out of 60 samples. RIN scores summarize the distribution of molecular weights and low RIN scores may confound further analyses. All four samples that failed showed a very low RIN score. Due to the use of a different purification method, we had no RIN scores available for the LCLs. Five successfully arrayed samples with high reproducibility between visits showed RIN scores between 1.5 and 6.5 (see Additional file 1). Hierarchical clustering showed however that isolation method was the major response variable and not RIN, yield, individual, chip, detection score or visit.

Probes were sequence matched to NCBI Build 36.1 (hg18) using the blastn algorithm to obtain a physical position from which Ensembl transcript and Gene identifiers were extracted. Probes that showed one mismatch or more were aligned to Ensembl transcripts or EMBL ESTs using BLAST (1), and genomic locations were then established by re-mapping the target transcript to genome (NCBI build 36) either by extracting annotation data from UCSC MySQL tables or by BLAST against genomic sequence. Probes overlapping at least 10 bases of repeat sequence, established by using RepeatMasker on the transcript sequence, were discarded. Probes with SNPs (minor allele frequency > 5%, http://www.hapmap.org) in their sequence or that had no match to the human genome build 36 were removed from the analysis. We could extract Ensembl transcripts identifiers for a total of 21,855 probes.

### Statistical analysis

For each method, data analysis was restricted to i) probes for which the detection score was greater than 95% in all samples or ii) probes with SD > 0.5 in all samples. We compared the number of detected probes between methods by using a McNemar test. For investigation of the biological reproducibility and the concordance between methods, we calculated spearman correlations between visits for each probe for each method. To compare biological reproducibility between two methods, we averaged the expression values of each probes across visits and calculated spearman correlations between methods.

For the clustering analysis, we used hierarchical clustering and PCA (using the NIPALS algorithm for estimating latent variables) on the normalised gene expression data of 7,305 probes that were detected across all 56 samples. In the PCA and PLS-DA analysis, the measurements of each expression probe were mean centered prior to the analysis. Using a PLS-DA model, we identified a set of transcripts that discriminates the method of interest from the other four methods. We computed a separate PLS-DA model for each method for which we set two classes as a response variable: one class for the method of interest and one class for the other four methods. We then extracted the w1 variable weights of the expression probes for each of the five PLS-DA models, ranked these variable weights and selected the 5% highest and 5% lowest ranked expression probes for each method. For a single vector, y, Trygg *et al. *suggested, that w1 should contain more useful interpretational information than the more commonly used regression coefficients [34].

To investigate the correlation between differentially expressed probes and cellular composition, we performed hierarchical clustering on the 374 differentially expressed probes. For each cluster of probes, we calculated spearman correlations between each probe by averaging the expression measurements of the two visits of the PAX samples and cell count parameters (neutrophils, lymphocytes and monocytes, mean cell volume and hemoglobin concentration). Subsequently, we compared the mean spearman correlations of the probes in each cluster with mean spearman correlation of all detected probes excluding the differentially expressed probes using a Wilcoxon rank-sum test. Multivariate analyses were performed using Evince (UmBio). All other analyses were conducted within the statistical scripting environment, R v2.4.1 [31].

### GO analysis

We investigated significant enrichment of specific GO terms among the set of probes that are specific for the method compared to the all probes detected for that specific method. In all GO analyses, Ensembl Gene Identifiers were tested using DAVID [35]. Enrichment of each GO term was evaluated through use of the Fisher's exact Test and corrected for multiple testing with FDR [36].

### Differential expression analysis

We used the Bioconductor R package Maanova to identify expression probes whose expression differed significantly between pairs of methods [37]. We fitted a linear mixed model for each probe using the Fs distribution as the null distribution and we fitted method as fixed, and visit and individual as random effects. We considered probes as differentially expressed when significant at a 5% FDR. We tested for significant enrichment of GO terms among the set of differentially expressed probes relative to the overlapping detected probes of two methods. Because a large proportion of probes were significantly differentially expressed, we selected the 5% of top hits ranked by FDR p-value. Of these 5% of top probes, we used only these probes that showed a more than a three fold change between methods.

## List of abbreviations

LCL: lymphoblastoid cell line; PBMC: Peripheral blood mononuclear cell; PAX: PAXgene; EBV: Epstein Barr virus; RIN: RNA Integrity Number; SD: standard deviation; PCA: Principal Components Analysis; PLS-DA: Partial Least Squares Discriminant Analysis; GO: gene ontology; FDR: false discovery rate.

## Authors' contributions

JM carried out data analysis and wrote the manuscript. MM conceived the study and its design. AB performed sample collection, cell purifications and RNA extractions. TW performed gene expression profiling. FP, HL, CL, JT, MA, KZ and MM contributed to the data analysis and interpretation of data. KZ and MM edited the manuscript. All authors read and approved the final manuscript.

## Supplementary Material

Additional file 1 RIN score for each method for 56 samples. Click here for file

Additional file 2 Overview of enrichment of Biological Processes of 5% highest and 5% lowest factor loadings across all 56 samples. * + = up-regulated probes; - down-regulated probes. Click here for file

Additional file 3 Enrichment of GO terms among uniquely expressed probes. Click here for file
